# Supplementary material for: A Klebsiella pneumoniae antibiotic resistance mechanism that subdues host defences and promotes virulence
Source: EMBO Mol Med. 2017 Feb 15;9(4):430–47. doi: 10.15252/emmm.201607336 (PMC5376759; doi:10.15252/emmm.201607336)
Supplement: Supplementary file 1 — Appendix [file EMMM-9-430-s001.pdf]

Appendix: A *Klebsiella pneumoniae* antibiotic resistance mechanism that subdues host defences and promotes virulence.

Timothy J Kidd, Grant Mills, Joana Sá-Pessoa, Amy Dumigan, Christian Frank, José L Insua, Rebecca Ingram, Laura Hobley and José A Bengoechea

Table of Contents

Appendix Figure S1. 2
Appendix Table S1. 3
Appendix Table S2. 4
Appendix Table S3. 5
Appendix Figure S2. 7
Appendix Figure S3. 8
Appendix Figure S4. 9
Appendix Figure S5. 10
Appendix Figure S6. 11
Appendix Figure S7. 12
Appendix Figure S8. 13
Appendix Table S4. 15
Appendix References. 16

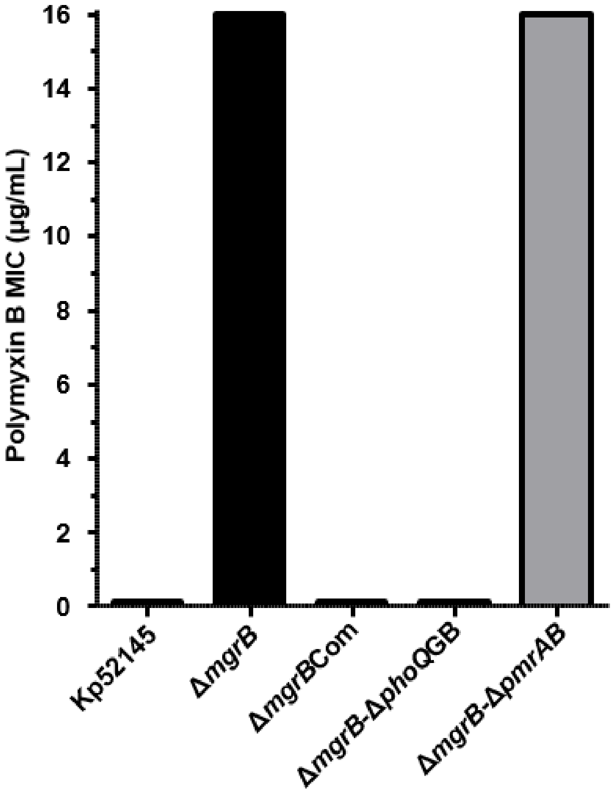

Appendix Figure S1.

Etest® minimal inhibitory concentrations (MIC) to polymyxin B of the *Klebsiella pneumoniae* 52145, 52145-Δ*mgrB*, 52145-Δ*mgrB*Com, 52145-Δ*mgrB*-Δ*phoQ*GB and 52145-Δ*mgrB*-Δ*pmrAB* strains.

Strain	Observed ion (*m/z*)	Acyl substitution	Proposed fatty acid, phosphate and carbohydrate composition[a]
52145 (wild-type)	1824	Hexa-acyl	4x C14:0(3-OH), 2x C14:0, 2P
	1840	Hexa-acyl	4x C14:0(3-OH), 1x C14:0, 1x C14:0(3-OH), 2P
	2063	Hepta-acyl	4x C14:0(3-OH), 2x C14:0, 1x C16:0, 2P
52145-Δ*mgrB*	1824	Hexa-acyl	4x C14:0(3-OH), 2x C14:0, 2P
	1840	Hexa-acyl	4x C14:0(3-OH), 1x C14:0, 1x C14:0(3-OH), 2P
	1852	Hexa-acyl	4x C14:0(3-OH), 1x C14:0, 1x C16:0, 2P
	1866	Hexa-acyl	4x C14:0(3-OH), 1x C14:0(3-OH), 1x C16:0, 2P
	1948	Hexa-acyl	4x C14:0(3-OH), 2x C14:0, 1x PEtN, 2P
	1955	Hexa-acyl	4x C14:0(3-OH), 2x C14:0, 1x Ara4N, 2P
	2063	Hepta-acyl	4x C14:0(3-OH), 2x C14:0, 1x C16:0, 2P
	2079	Hepta-acyl	4x C14:0(3-OH), 1x C14:0, 1x C14:0(3-OH), 1x C16:0, 2P
52145-Δ*mgrB*Com	1824	Hexa-acyl	4x C14:0(3-OH), 2x C14:0, 2P
	1840	Hexa-acyl	4x C14:0(3-OH), 1x C14:0, 1x C14:0(3-OH), 2P
	2063	Hepta-acyl	4x C14:0(3-OH), 2x C14:0, 1x C16:0, 2P
52145-Δ*mgrB*-Δ*phoQ*GB	1797	Hexa-acyl	4x C14:0(3-OH), 1x C14:0, 1x C12:0, 2P
	1824	Hexa-acyl	4x C14:0(3-OH), 2x C14:0, 2P
	1840	Hexa-acyl	4x C14:0(3-OH), 1x C14:0, 1x C14:0(3-OH), 2P
	2063	Hepta-acyl	4x C14:0(3-OH), 2x C14:0, 1x C16:0, 2P
52145-Δ*mgrB*-Δ*pmrAB*	1824	Hexa-acyl	4x C14:0(3-OH), 2x C14:0, 2P
	1840	Hexa-acyl	4x C14:0(3-OH), 1x C14:0, 1x C14:0(3-OH), 2P
	1852	Hexa-acyl	4x C14:0(3-OH), 1x C14:0, 1x C16:0, 2P
	1866	Hexa-acyl	4x C14:0(3-OH), 1x C14:0(3-OH), 1x C16:0, 2P
	1948	Hexa-acyl	4x C14:0(3-OH), 2x C14:0, 1x PEtN, 2P
	1955	Hexa-acyl	4x C14:0(3-OH), 2x C14:0, 1x Ara4N, 2P
	2063	Hepta-acyl	4x C14:0(3-OH), 2x C14:0, 1x C16:0, 2P
	2079	Hepta-acyl	4x C14:0(3-OH), 1x C14:0, 1x C14:0(3-OH), 1x C16:0, 2P
52145-Δ*mgrB*-Δ*phoQ*GB-Δ*pmrAB*	1797	Hexa-acyl	4x C14:0(3-OH), 1x C14:0, 1x C12:0, 2P
	1824	Hexa-acyl	4x C14:0(3-OH), 2x C14:0, 2P
	1840	Hexa-acyl	4x C14:0(3-OH), 1x C14:0, 1x C14:0(3-OH), 2P
	2063	Hepta-acyl	4x C14:0(3-OH), 2x C14:0, 1x C16:0, 2P
52145-Δ*mgrB*-Δ*phoQ*GB-*phoPQ*Com	1824	Hexa-acyl	4x C14:0(3-OH), 2x C14:0, 2P
	1840	Hexa-acyl	4x C14:0(3-OH), 1x C14:0, 1x C14:0(3-OH), 2P
	1852	Hexa-acyl	4x C14:0(3-OH), 1x C14:0, 1x C16:0, 2P
	1866	Hexa-acyl	4x C14:0(3-OH), 1x C14:0(3-OH), 1x C16:0, 2P
	1948	Hexa-acyl	4x C14:0(3-OH), 2x C14:0, 1x PEtN, 2P
	1955	Hexa-acyl	4x C14:0(3-OH), 2x C14:0, 1x Ara4N, 2P
	2063	Hepta-acyl	4x C14:0(3-OH), 2x C14:0, 1x C16:0, 2P
	2079	Hepta-acyl	4x C14:0(3-OH), 1x C14:0, 1x C14:0(3-OH), 1x C16:0, 2P

[a] C14:0(3-OH), R-3-hydroxymyristoyl acyl chain; C14:0, myristate acyl chain; P, phosphate; C16:0, palmitate; PEtN, phosphoethanolamine; Ara4N, 4-amino-4-deoxy-l-arabinose; C12:0, laureate.

Appendix Table S1.

Lipid A species identified by negative-ion MALDI-TOF mass spectrometry analysis among the *Klebsiella pneumoniae* 52145 strains when grown in LB medium at 37^{0}C.

Strain[a]	Origin	Colistin susceptibility	*mgrB* sequence mutation[b]	Additional antimicrobial resistance determinants[c]	Multilocus sequence type
T1a	Turkey	Sensitive	Wild-type	KPC-2	ST-258
T1b	Turkey	Resistant	Truncated by IS5-like	KPC-2	ST-258
C21	Colombia	Resistant	Truncated by ISKpn13	CTX-M-2	ST-1271
C22	Colombia	Resistant	Truncated by ISKpn14	CTX-M-15	ST-101
C2	Colombia	Resistant	Truncated by IS10R	KPC-2	ST-258
15I5	France	Resistant	Truncated protein (29 aa)	-	ST-70

[a] Strain designation and isolates were identical to those reported by Poirel *et al* (Poirel et al, 2015)

[b] IS, insertion sequence, aa, amino acid

[c] KPC, *Klebsiella pneumoniae* carbapenemase 2; CTX-M, CTX-M extended spectrum beta-lactamase

Appendix Table S2.
Origins, characteristics and genotypes of the clinical *Klebsiella pneumoniae* strains used in this study.

Bacterial strain or plasmid	Genotype or comments[a]	Source or reference(s)
Strains		
Escherichia coli		
C600	*thi thr leuB tonA lacY supE*	
GT115	F^- *mcrA*Δ(*mrr-hsdRMS-mcrBC*) ϕ80Δ*lacZ*ΔM15 Δ*lacX*74 *recA1rpsL* (StrA) *endA1*Δ*dcm uidA(*Δ*MluI)::* *pir-116* Δ*sbcC-sbcD*	InvivoGen
SY327	*λ (lac pro) argE (Am) rif nalA recA56 (λ pir)*	Miller, 1988
β2163	(F^-) RP4-2-Tc::Mu Δ*dapA*::(*erm-pir*) [$Km^R Em^R$]	Demarre, 2005
Klebsiella pneumoniae		
52145	Clinical isolate; serotype O1:K2; sequence type ST66	Brisse, 2009; Lery, 2014
52145-Δ*mgrB*	Kp52145, Δ*mgrB*; the *mgrB* gene was inactivated	This study
52145-Δ*mgrB*Com	52145-Δ*mgrB* complemented with mini-Tn7TKmKp52145*mgrB*; *mgrB* activity was restored	This study
52145-Δ*pmrC*	Kp52145, Δ*pmrC*; the *pmrC* gene was inactivated	This study
52145-Δ*pagP*	Kp52145, Δ*pagP*; the *pagP* gene was inactivated	This study
52145-Δ*manC*	Kp52145, Δ*manC*; the *manC* gene was inactivated; no CPS expression	This study
52145-Δ*mgrB*-Δ*phoQ*GB	Kp52145, Δ*mgrB*, Δ*phoQ*::KM-GenBlock; the *mgrB* and *phoQ* genes were inactivated; Km^R	This study
52145-Δ*mgrB*-Δ*pmrAB*	Kp52145, Δ*mgrB*, Δ*pmrAB*; the *mgrB* and *pmrAB* genes were inactivated	This study
52145-Δ*mgrB*-Δ*phoQ*GB-*phoPQ*Com	52145-Δ*mgrB*-Δ*phoQ*GB complemented with pGP-Tn7-Cm_KpnPhoPQCom; *phoPQ* activity was restored	This study
52145-Δ*mgrB*-Δ*lpxO*	Kp52145, Δ*mgrB*, Δ*lpxO*; the *mgrB* and *lpxO* genes were inactivated	This study
52145-Δ*pagP*-Δ*mgrB*	Kp52145, Δ*pagP*, Δ*mgrB*; the *pagP* and *mgrB* genes were inactivated	This study
52145-Δ*pmrC*-Δ*mgrB*	Kp52145, Δ*pmrC*, Δ*mgrB*; the *pmrC* and *mgrB* genes were inactivated	This study
52145-Δ*mgrB*-Δ*pmrF*	Kp52145, Δ*mgrB*, Δ*pmrF*; the *mgrB* and *pmrF* genes were inactivated	This study
52145-Δ*mgrB*-Δ*manC*	Kp52145, Δ*mgrB*, Δ*manC*; the *mgrB* and *manC* genes were inactivated; no CPS expression	This study
52145-Δ*pmrC*-Δ*lpxO*-Δ*mgrB*	Kp52145, Δ*pmrC*, Δ*lpxO*, Δ*mgrB*; the *pmrC*, *lpxO* and *mgrB* genes were inactivated	This study
52145-Δ*mgrB*-Δ*lpxO*-Δ*pmrF*	Kp52145, Δ*mgrB*, Δ*lpxO*, Δ*pmrF*; the *mgrB*, *lpxO* and *pmrF* genes were inactivated	This study
52145-Δ*pmrC*-Δ*lpxO*-Δ*mgrB*-Δ*pmrF*	Kp52145, Δ*pmrC*, Δ*lpxO*, Δ*mgrB*, Δ*pmrF*; the *pmrC*, *lpxO*, *mgrB* and *pmrF* genes were inactivated	This study
52145-Δ*pmrC*-Δ*lpxO*-Δ*mgrB*-Δ*pmrF*-Δ*pagP*	Kp52145, Δ*pmrC*, Δ*lpxO*, Δ*mgrB*, Δ*pagP*; the *pmrC*, *lpxO*, *mgrB*, *pmrF* and *pagP* genes were inactivated	This study
52145-Δ*mgrB*-Δ*lpxO*-*lpxO*Com	52145-Δ*mgrB*Δ*lpxO* complemented with pGP-Tn7-Cm_KpnLpxOCom; *lpxO* activity was restored	This study

Continued

Plasmids		
pGEM-T Easy	Cloning plasmid; Amp^{R}	Promega
pGPI-SceI-2	Suicide vector, R6Kɣ origin of replication, Mob^{+}, carries a I-SceI endonuclease site; Tmp^{R}	Aubert, 2014
pDAI-SceI-SacB	Expresses the I-SceI endonuclease, *sacB* gene; Tet^{R}	Aubert, 2014
pKD4	PCR template plasmid for the λ Red recombinase system; Km^{R}	Datsenko, 2000
pKOBEG-sacB	λ phage *red*γβα, arabinose inducible; Cm^{R}	Derbise, 2003
pTSNSK-Tp	pTSNSK-Tp containing a transposase for Tn7 insertion; Km^{R}, Tmp^{R}	Crépin, 2012
pUC18R6KT-mini-Tn7TKm	pUC18R6KT-mini-Tn7T complementation vector; Amp^{R}, Km^{R}	Choi, 2005
pGP-Tn7-Cm	pGP-Tn7 complementation vector; Amp^{R}, Cm^{R}	Crépin, 2012
pMAKSACB	Suicide vector, Psc101 replication origin, Mob+, *sacB* gene; Cm^{R}	Favre, 2000
pFLP2	Plasmid encoding FLP to remove cassettes between FRT sites, *sacB* gene; Tmp^{R}	Hoang, 1998
pGPL01	Firefly luciferase (*lucFF*) transcriptional fusion suicide vector, R6Kɣ origin of replication; Amp^{R}	Gunne, 1996
pGEMΔ*mgrB*	pGEM-T Easy containing Δ*mgrB*; Amp^{R}	This study
pGEMΔ*pmrAB*	pGEM-T Easy containing Δ*pmrAB*; Amp^{R}	This study
pGEMΔ*manC*	pGEM-T Easy containing Δ*manC*; Amp^{R}	This study
pGEMΔ*pmrF*	pGEM-T Easy containing Δ*pmrF*; Amp^{R}	This study
pGEMΔ*pagP*	pGEM-T Easy containing Δ*pagP*; Amp^{R}	This study
pGPI-SceI-2Δ*mgrB*	pGPI-SceI-2 containing Δ*mgrB*; Tmp^{R}	This study
pGPI-SceI-2Δ*pmrAB*	pGPI-SceI-2 containing Δ*pmrAB*; Tmp^{R}	This study
pGPI-SceI-2Δ*manC*	pGPI-SceI-2 containing Δ*manC*; Tmp^{R}	This study
pGPI-SceI-2Δ*pmrF*	pGPI-SceI-2 containing Δ*pmrF*; Tmp^{R}	This study
pGPI-SceI-2Δ*pagP*	pGPI-SceI-2 containing Δ*pagP*; Tmp^{R}	This study
pMAKSACBΔ*lpxO*	pMAKSACB containing Δ*lpxO*; Km^{R}	This study
pUC18R6KT-mini-Tn7TKmKp52145*mgrB*	pUC18R6KT-mini-Tn7TKm containing *mgrB* gene for complementation; Amp^{R}, Km^{R}	This study
pGP-Tn7-Cm_KpnPhoPQCom	pGP-Tn7-Cm containing *phoPQ* operon for complementation; Amp^{R}, Cm^{R}	Llobet, 2015
pGP-Tn7-Cm_KpnLpxOCom	pGP-Tn7-Cm containing *lpxO* gene for complementation; Amp^{R}, Cm^{R}	Llobet, 2015
pGPLKpnProLpxO	pGPL01 containing the *lpxO* promoter region; Amp^{R}	Llobet, 2015
pGPLKpnProPmrH	pGPL01 containing the *pmrH* promoter region; Amp^{R}	Llobet, 2011
pGPLKpnProPmrC	pGPL01 containing the *pmrC* promoter region; Amp^{R}	Insua, 2013
pGPLKpnProPagP	pGPL01 containing the *pagP* promoter region; Amp^{R}	This study
pGPLKpnProPhoP	pGPL01 containing the *phoP* promoter region; Amp^{R}	Llobet, 2011
pGPLKpnProcps	pGPL01 containing the *cps* promoter region; Amp^{R}	Llobet, 2011

[a] Km^{R}, kanamycin resistant; Em^{R}, erythromycin resistant; Tmp^{R}, trimethoprim resistant; Amp^{R}, ampicillin resistant; Tet^{R}, tetracycline resistant; Cm^{R}, chloramphenicol resistant

Appendix Table S3.

Bacterial strains and plasmids used in this study

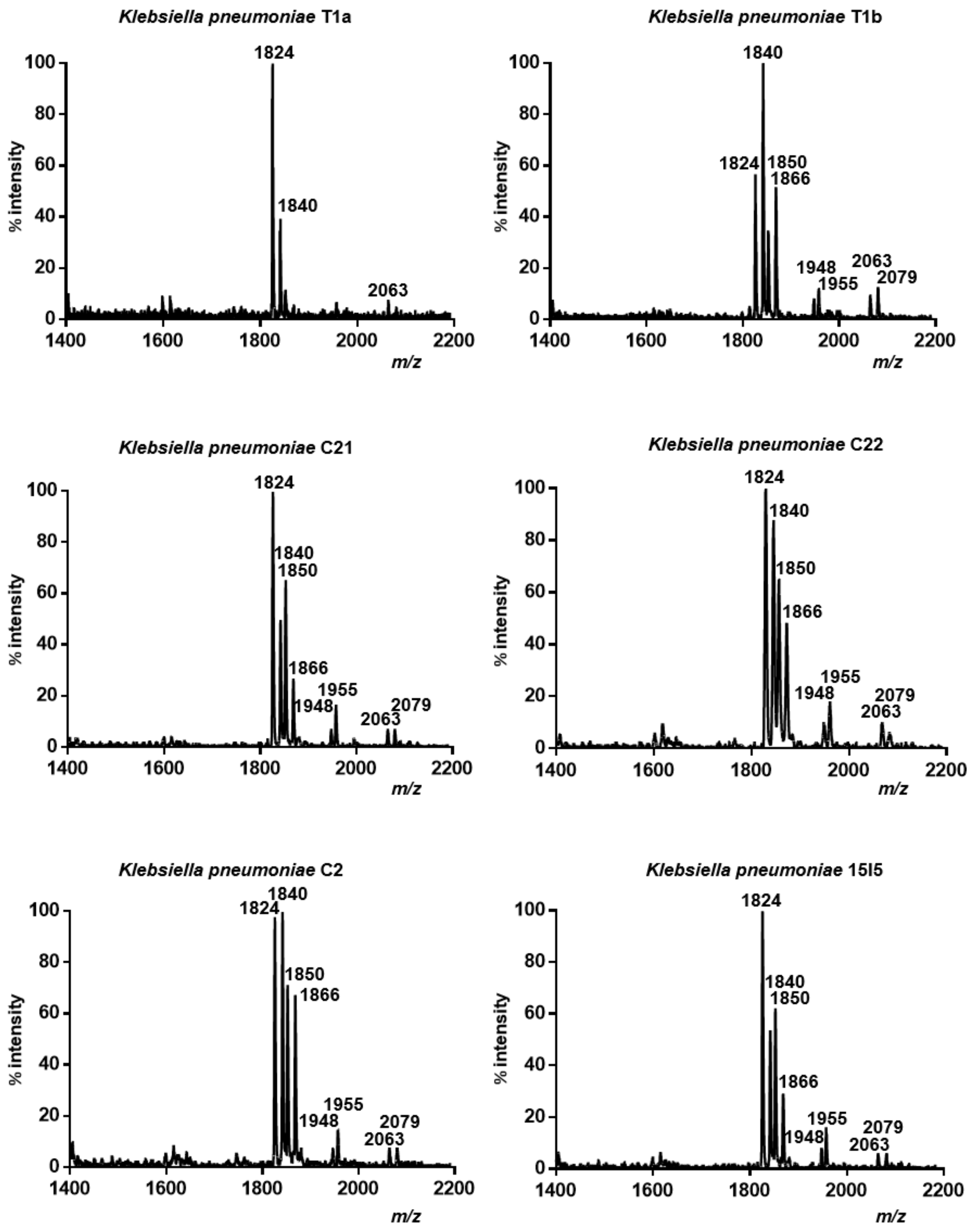

Appendix Figure S2.

Negative ion MALDI-TOF mass spectrometry spectra of lipid A isolated from the clinical *Klebsiella pneumoniae* T1a (A), T1b (B), C21 (C), C22 (D), C2 (E) and 15I5 (F) strains. Data represent the mass to charge (*m/z*) ratios of each lipid A species detected and were representative of three independent lipid A extractions.

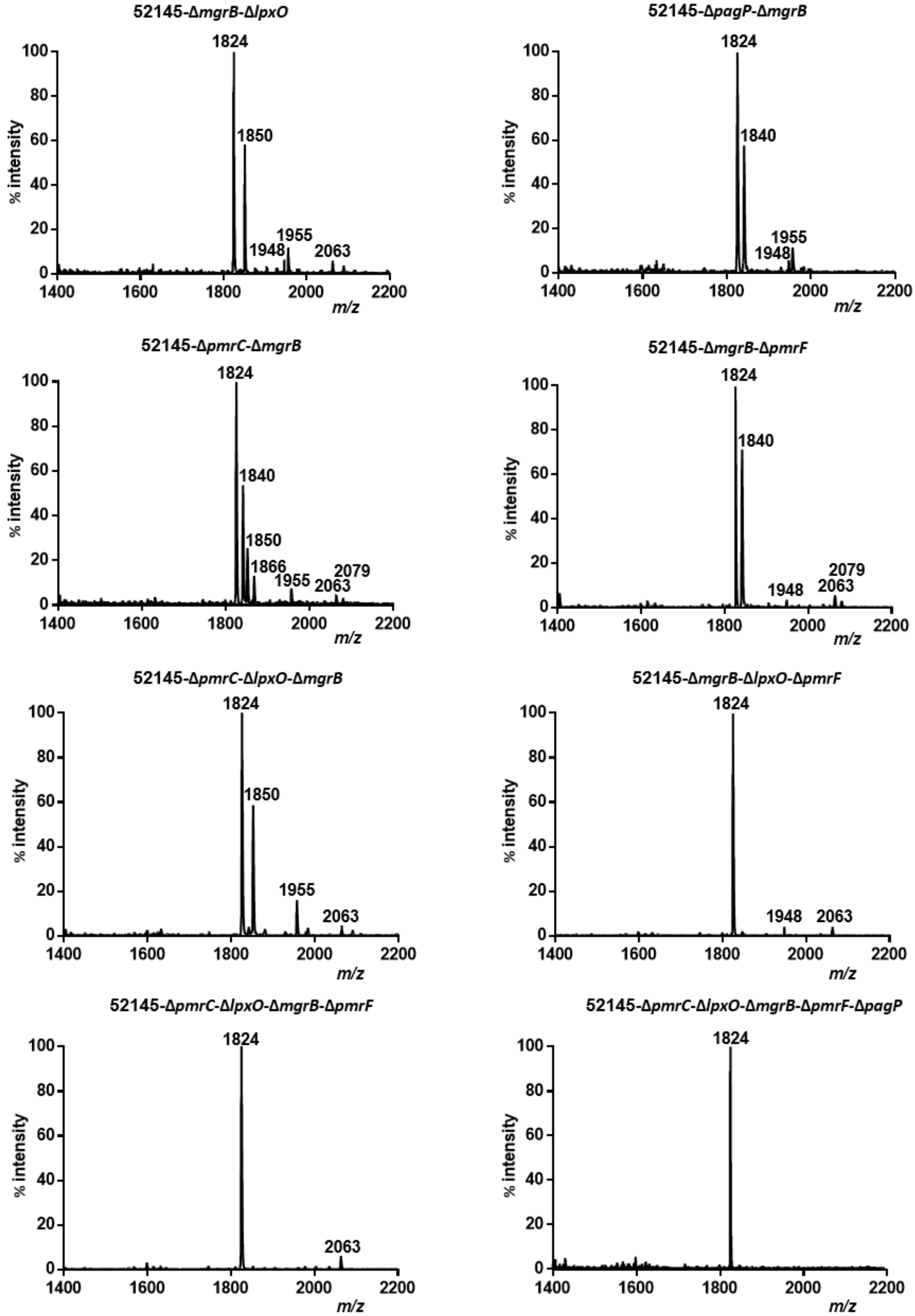

Appendix Figure S3.

Negative ion MALDI-TOF mass spectrometry spectra of lipid A isolated from the *Klebsiella pneumoniae* 52145-Δ*mgrB*-Δ*lpxO*, 52145-Δ*pagP*-Δ*mgrB*, 52145-Δ*pmrC*-Δ*mgrB*, 52145-Δ*mgrB*-Δ*pmrF*, 52145-Δ*pmrC*-Δ*lpxO*-Δ*mgrB*, 52145-Δ*mgrB*-Δ*lpxO*-Δ*pmrF*, 52145-Δ*pmrC*-Δ*lpxO*-Δ*mgrB*-Δ*pmrF* and 52145-Δ*pmrC*-Δ*lpxO*-Δ*mgrB*-Δ*pmrF*-Δ*pagP* strains. Data represent the mass to charge (*m/z*) ratios of each lipid A species detected and were representative of three independent lipid A extractions.

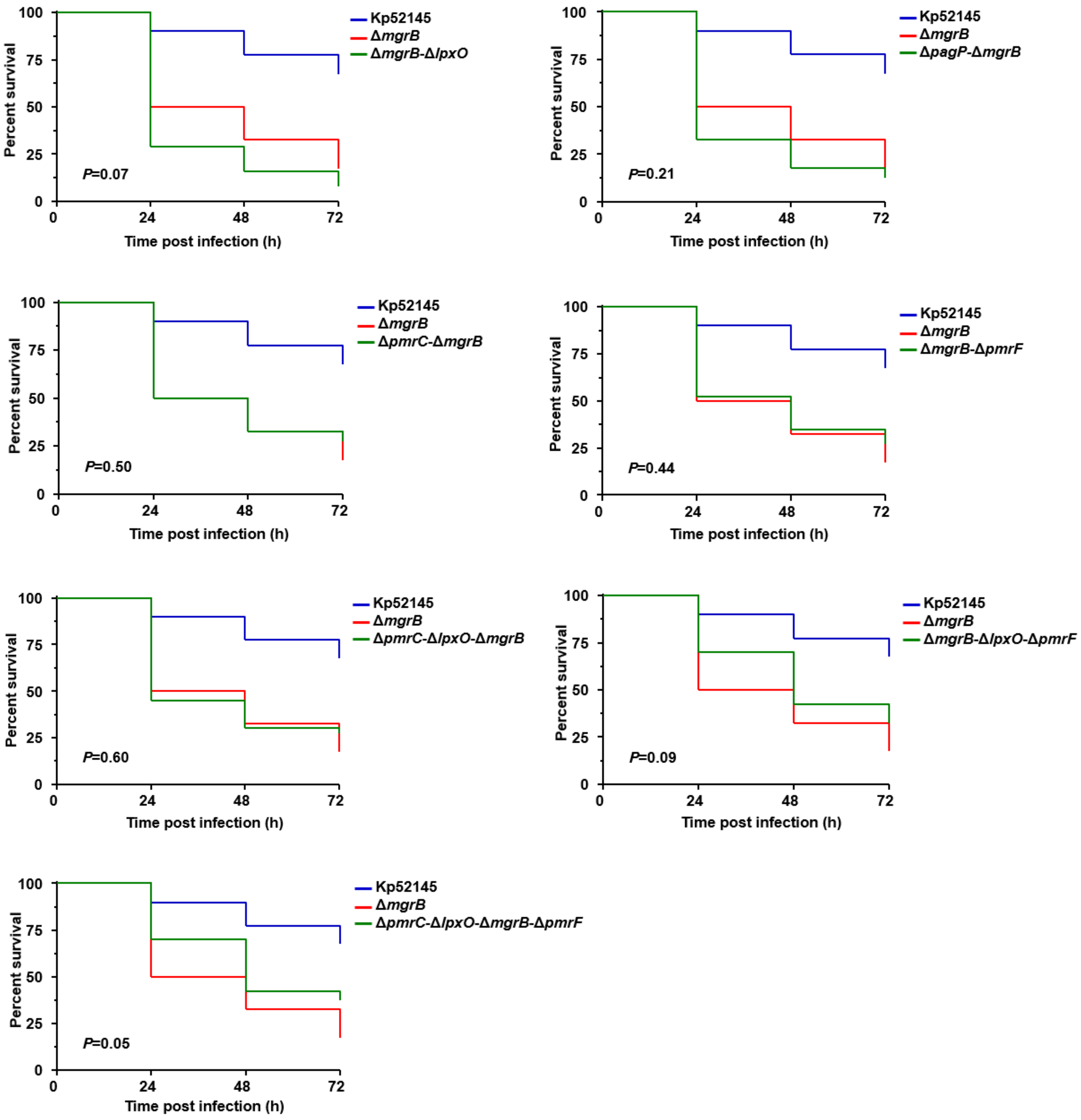

Appendix Figure S4.

Kaplan-Meier plots showing the percent survival of *Galleria mellonella* over 72 hours post-infection with 10^5 organisms of *Klebsiella pneumoniae* 52145 (blue), 52145-Δ*mgrB* (red), and the seven double, triple and quadruple mutant (green) strains. Forty larvae were infected in each group. Level of significance was determined using the log-rank (Mantel-Cox) test with Bonferroni correction (α=0.017). *P*-values presented correspond to the difference between 52145-Δ*mgrB* and the various multiple mutant strains.

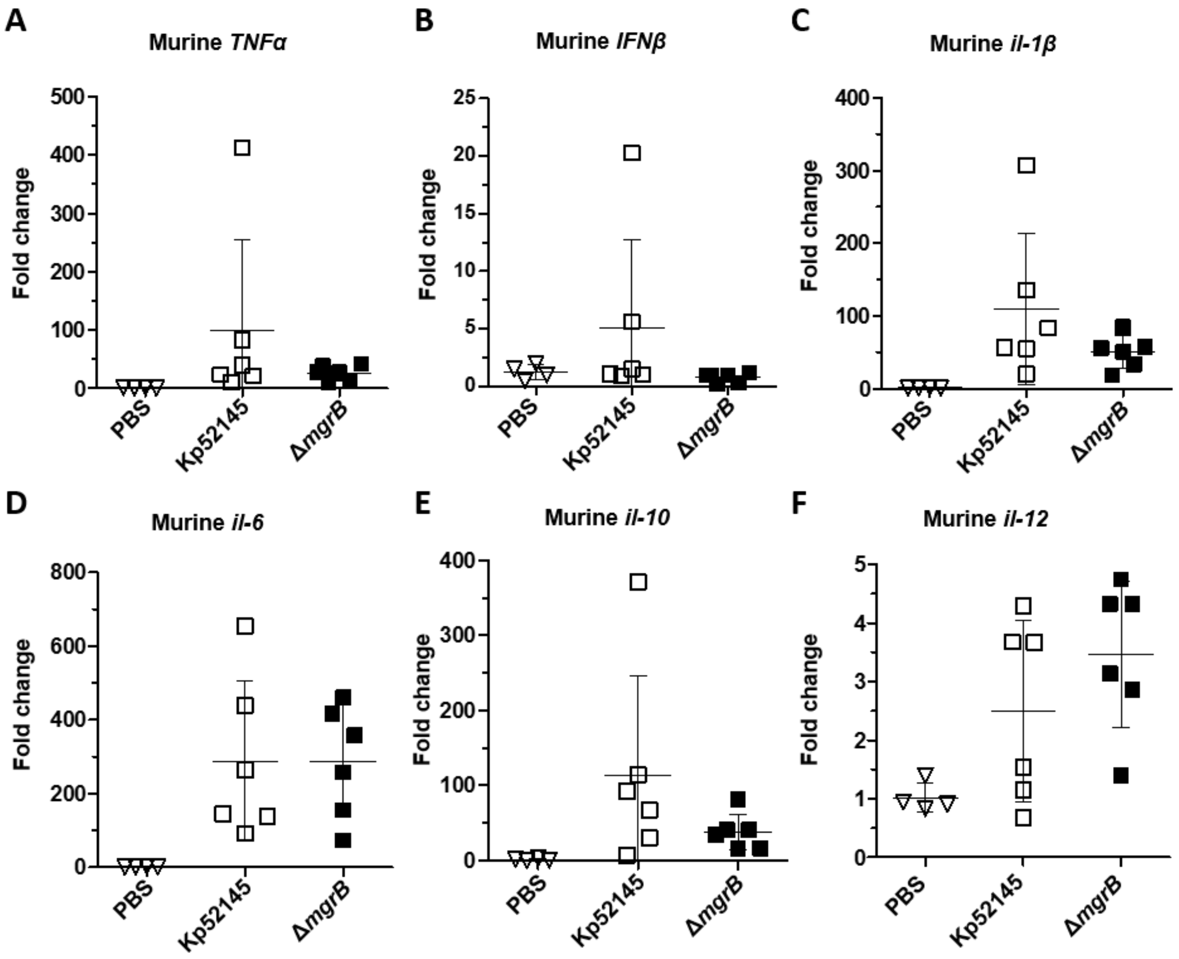

Appendix Figure S5.

Expression of murine tissue necrosis factor-alpha (*TNFα*; A), interferon-beta (*IFNβ*; B), interleukin [il] 1-beta (*il-1β*; C), *il-6* (D), *il-10* (E) and *il-12* (F) produced in the lung after 24 hours infection with *Klebsiella pneumoniae* 52145 and 52145-Δ*mgrB* as determined by reverse transcriptase quantitative real-time PCR (n=4 PBS vehicle controls, n=6 infected mice per group). Values are presented as the mean ± SD of two independent cDNA preparations measured in duplicate. Level of significance versus Kp52145 was determined using the two-way Unpaired t-test.

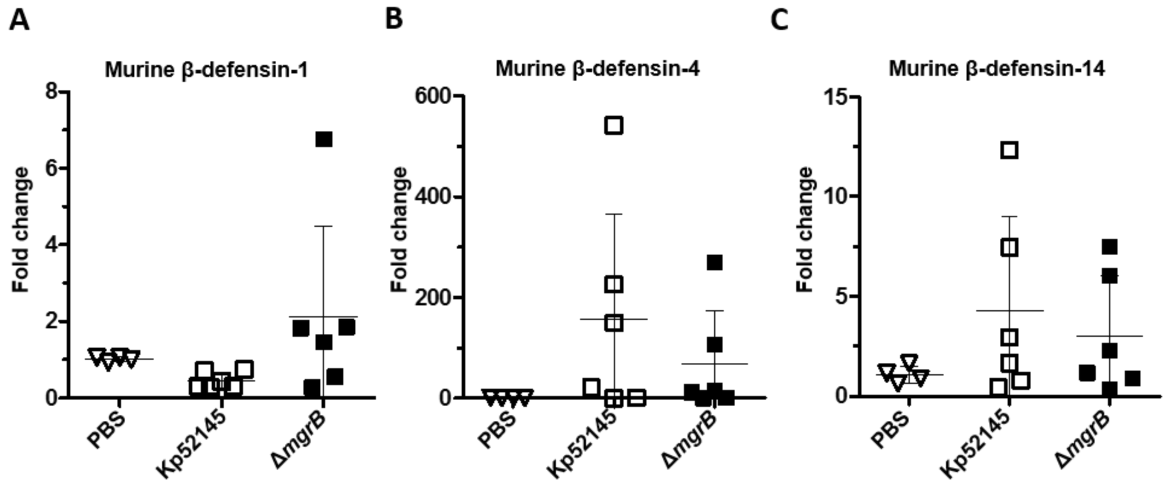

Appendix Figure S6.

Expression of the murine antimicrobial peptides beta defensin-1 (A), beta defensin-4 (B) and beta defensin-14 (C) produced in the lung after 24 hours infection with 52145 and 52145-Δ*mgrB* as determined by reverse transcriptase quantitative real-time PCR (n=4 PBS vehicle controls, n=6 infected mice per group). Values are presented as the mean ± SD of two independent cDNA preparations measured in duplicate. Level of significance versus Kp52145 was determined using the two-way Unpaired t-test.

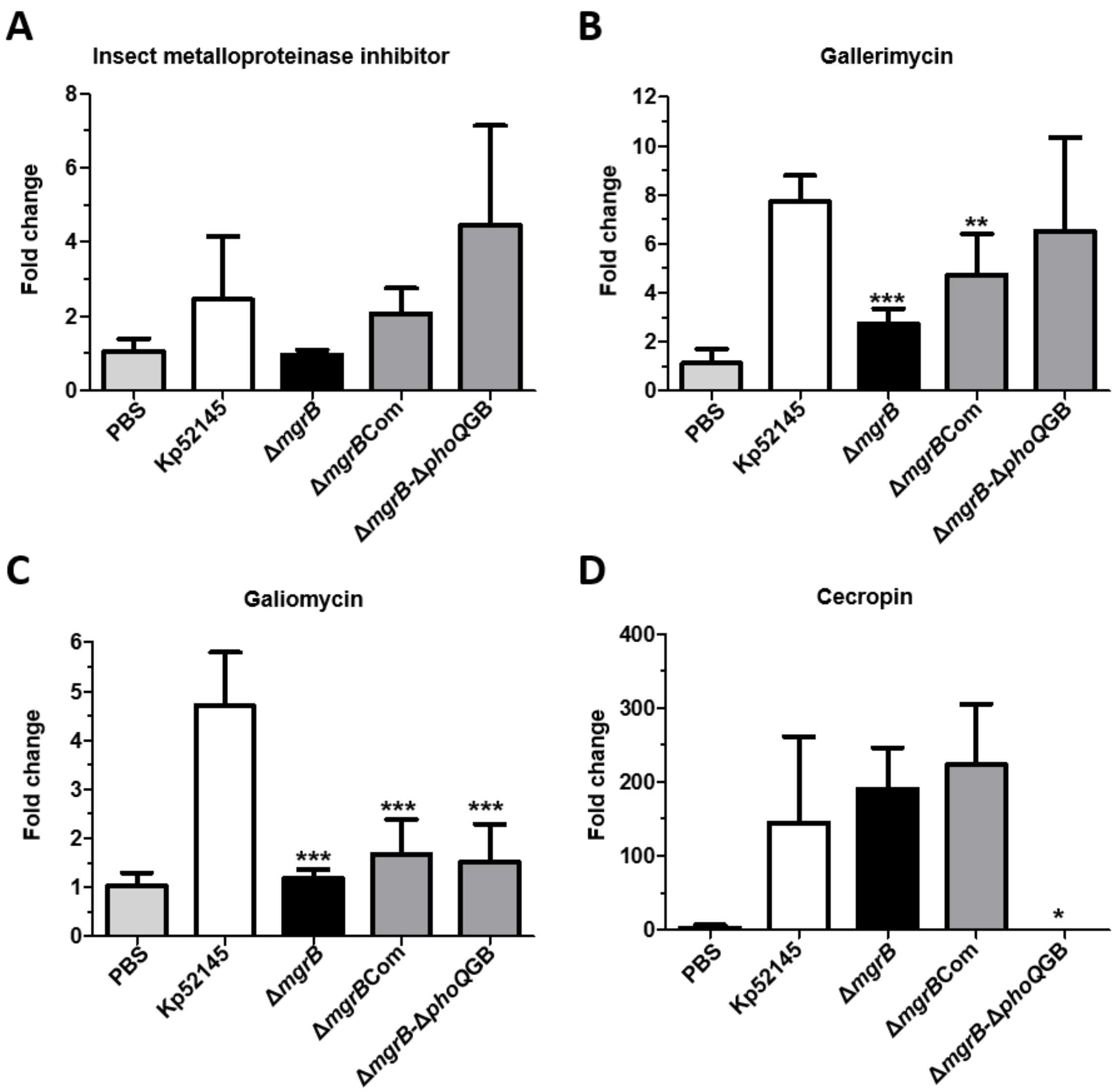

Appendix Figure S7.

Expression of insect metalloproteinase inhibitor (A), gallerimycin (B), galiomycin (C) and cecropin (D) produced by *Galleria mellonella* after eight hours infection with *Klebsiella pneumoniae* 52145, 52145-Δ*mgrB*, 52145-Δ*mgrB*Com and 52145-Δ*mgrB*-Δ*phoQ*GB as determined by reverse transcriptase quantitative real-time PCR. Three larvae per group were infected and values are presented as the mean ± SD of two independent cDNA preparations measured in duplicate. *, P=0.01; **, P=0.004, ***, P≤0.0002; versus Kp52145 determined using the two-way Unpaired t-test.

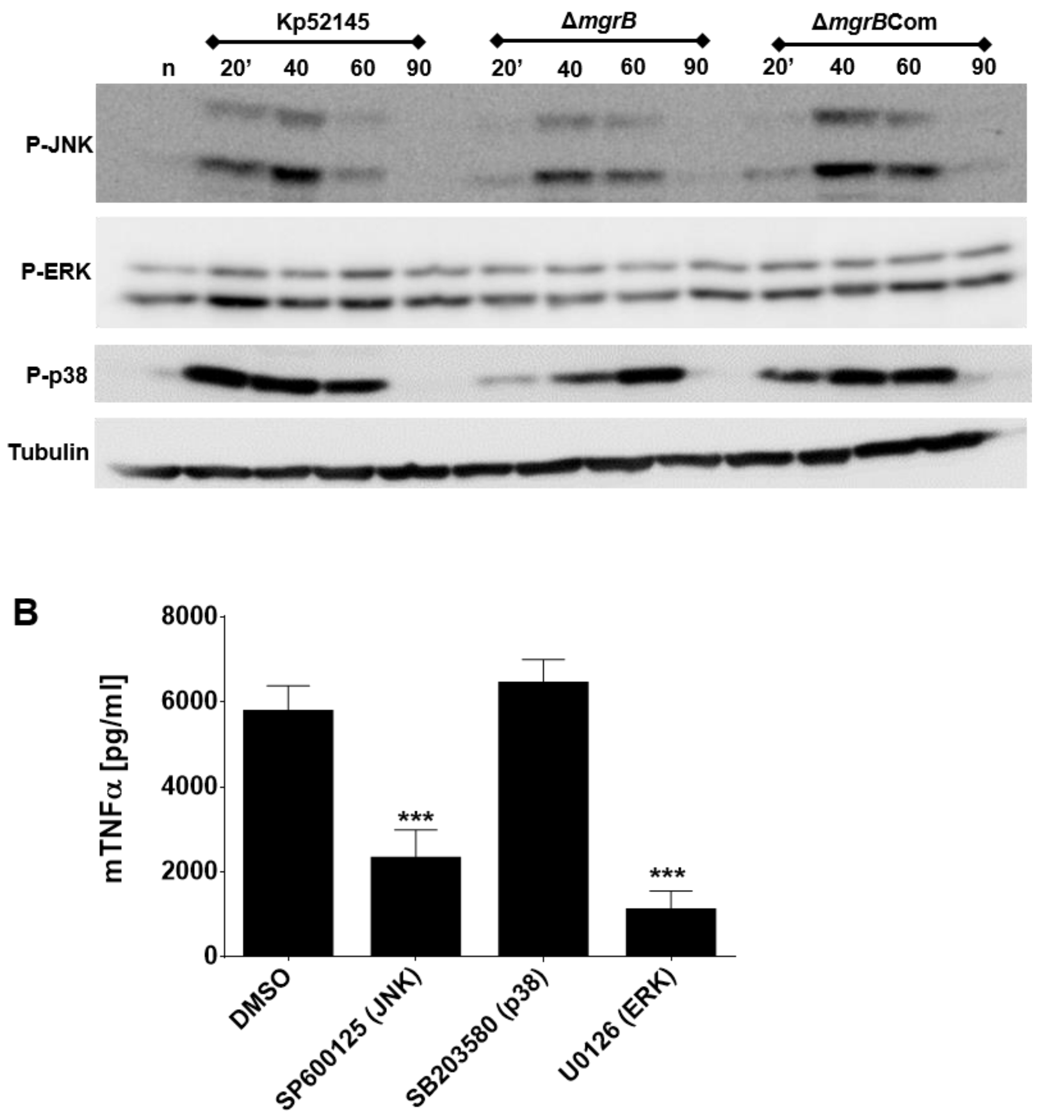

Appendix Figure S8.

(A) Early inflammatory responses were analysed by immunoblot of phospho-ERK (P-ERK), phospho-p38 (P-p38), phospho-JNK (P-JNK) and tubulin levels in lysates of MH-S cells infected with *K. pneumoniae* 52145, 52145-Δ*mgrB*, 52145-Δ*mgrB*Com for the indicated times. (B) TNFα secretion upon *K. pneumoniae* infection in iBMDM macrophages was analysed in the presence of the MAPK ERK inhibitor (U0126, 10 μM, 2h before infection), JNK inhibitor (SP600125, 10 μM, 2 hours before infection), p38 inhibitor (SB203580, 10 μM, 2 hours before infection) or DMSO (vehicle solution). Macrophages were stimulated for 6 hours with 1 x 10^5 UV-killed *K. pneumoniae* 52145 and TNFα levels were determined in the supernatant of infected cells. ***, *P*<0.0001; versus DMSO control determined using the two-way Unpaired t-test.

Primer Name	Sequence (5'-3')[a]
*mgrB*_UPFWD	ATA TCG CCC ATT CGT TGC CT
*mgrB*_UPRVS	GGA TCC CCG TGG TAA GAC ATT TTT CTG CC
*mgrB*_DWNFWD	GGA TCC TTC ACT CCA CTC TCC GCA TC
*mgrB*_DWNRVS	GGC TAA GCA TCT CTT TCC CA
*pmrC*_FWD	GAT CTC ATT GGT GTC GCA GCT CGG CGT GAA TTT GCG AAA CGT GTA GGC TGG AGC TGC TTC
*pmrC*_RVS	CGA AGA TGC CAC GCA GAA AGC GGT GAT GCA TAA CGC TCC CCA TAT GAA TAT CCT CCT TAG
*pagP*_FWD	TGT CCG GAA ACG CCA GCG CGT CGT TTT CAT CGA CCC TTA GGT GTA GGC TGG AGC TGC TTC
*pagP*_RVS	AAG TAA ACT TAC CGT TAT TGT AGG TGC CGG GAA TAT AGG TCA TAT GAA TAT CCT CCT TAG
*pmrC*_checkFWD	CCT TCA CTT TGC ATC GCC AG
*pmrC*_checkRVS	CCT GTT CAT TCT GCT CAG C
*pagP*_checkFWD	TTA AGC CTC CGA CGA ACA GG
*pagP*_checkRVS	GCA CAA GAC CTT CCG CTT AT
*lpxO*_UPFWD	CCC AGG CGC AGA TTG CCC AG
*lpxO*_UPRVS	GGA TCC CGG ATC CGG ACT CAC TAT AGG GCG GAT ATT GAA CGG CCG ATG
*lpxO*_DWNFWD	GGA TCC CGG ATC CGG ACT CAC TAT AGG GGC GGT AAA TGT GGA ATG GTC G
*lpxO*_DWNRVS	TCC GTT CAC TGC GTG CCC TG
*pmrAB*_UPFWD	CGA TCC TGG ATG CGG ATA AA
*pmrAB*_UPRVS	GGA TCC AAC GTC AGC ACC CAT TGA
*pmrAB*_DWNFWD	GGA TCC CGC ACA CGT ATC CTT CAC TT
*pmrAB*_DWNRVS	CAG AAC GTC ACC GAC TTG AA
*pmrF*_UPFWD	ACC CAT TAT AAA GGC CGC CA
*pmrF*_UPRVS	GGA TCC GGC AGG CTA TCC TGT TCG TT
*pmrF*_DWNFWD	GGA TCC TCG GCC GCA TCT ATA ACG AC
*pmrF*_DWNRVS	GTG CAC ATG CCG TAC ACT TC
*pagP*_UPFWD	CGG CTT CGA CGC TAA TTT
*pagP*_UPRVS	GGA TCC AGG TAT GGT AAC CTT CGC TA
*pagP*_DWNFWD	GGA TCC TTC CCG GCA CCT ACA ATA AC
*pagP*_DWNRVS	ATC CTG TGC ACC ATG CTA TC
*manC*_UPFWD	TCG AAA GTG CTG AGT GGT CC
*manC*_UPRVS	GGA TCC TCA AGC GGT GCT CTT CGT TAC AG
*manC*_DWNFWD	GGA TCC TAA CTG ACC GAG AAC CAG TCC AC
*manC*_DWNRVS	GCC TCG TCA TCG AAC AGG AAG
PhoPQ_checkFWD	ATC CTG ATG GCT GAC AAG GC
PhoPQ_checkRVS	TCG GGG ATA AAC GGT AGT GG
*lpxO*_checkFWD	CTC GGG GTT GTA CCA GAT CG

Continued

*lpxO*_checkRVS	TTA ACG GCG GCT TTT TCC TG
*pagP*_Pro_FWD	ATC GAA TTC GCT GAT ACC GAC ATT CAC CA
*pagP*_Pro_RVS	GGA TCC CTT CGC TAA GGG TCG ATG AAA
*lucFF*_checkFWD	GAA GCG AAG GTT GTG GAT CT
*lucFF*_checkRVS	AAC CGG GAG GTA GAT GAG AT
*pagP*_Pro_checkFWD	GTC GTT GAC ATC GAG CAT CT
*phoP*_Pro_checkRVS	CAG CGC CGG TTC GAT ATA AA
*lpxO*_Pro_checkFWD	CTG TCC CGT GAA TTC TCC AT
*pmrH*_Pro_checkFWD	CGG CTG GAA CTA CGA GAT TTA C
*pmrC*_Pro_checkRVS	CGT CGG CGA TGT TTG AGT AT
*cps*_Pro_checkRVS	CTA TCT TCC TGT GGC TGC TC
mhPRT-FWD	GAT CAG TCA ACG GGG GAC AT
mHPRT-RVS	GGT CCT TTT CAC CAG CAA GC
mTNFα-FWD	TTC TGT CTA CTG AAC TTC GGG GTG ATC GGT CC
mTNFα-RVS	GTA TGA GAT AGC AAA TCG GCT GAC GGT GTG GG
mIFNβ-FWD	ATG GTG GTC CGA GCA GAG AT
mIFNβ-RVS	CCA CCA CTC ATT CTG AGG
mil-1β-FWD	AGA TGA AGG GCT GCT TCC AAA
mil-1β-RVS	AAT GGG AAC GTC ACA GAC CA
mil-6-FWD	ATG GAT GCT ACC AAA CTG GAT
mil-6-RVS	TGA AGG ACT CTG GCT TTG TCT
mil-10-FWD	GGA CTT TAA GGG TTA CTT GGG TTG CC
mil-10-RVS	CAT GTA TGC TTC TAT GCA GTT GAT GA
mil-12-FWD	GGA AGC ACG GCA GCA GAA TA
mil-12-RVS	AAC TTG AGG GAG AAG TAG GAA TGG
mβ-defensin-1-FWD	AAC ACG GTA CAC AGG CTT CC
mβ-defensin-1-RVS	TCA CAG ATG TCC AAG TCC CA
mβ-defensin-4-FWD	CTC CAC TTG CAG CCT TTA CC
mβ-defensin-4-RVS	GTG CAT CCC CTA GAA CTG GA
mβ-defensin-14-FWD	ATC TTG TTC TTG GTG CCT GC
mβ-defensin-14-RVS	CTT CTT TCG GCA GCA TTT TC

[a] Underlined text denotes a BamHI restriction site incorporated into the primer

Appendix Table S4.

Oligonucleotide primers used in this study

Appendix References.

Aubert D, Hamad M, Valvano M (2014) A markerless deletion method for genetic manipulation of *Burkholderia cenocepacia* and other multidrug-resistant gram-negative bacteria. In Host-Bacteria Interactions, Vergunst AC, O'Callaghan D (eds) pp 311-327. Springer New York

Brisse S, Fevre C, Passet V, Issenhuth-Jeanjean S, Tournebize R, Diancourt L, Grimont P (2009) Virulent clones of *Klebsiella pneumoniae*: identification and evolutionary scenario based on genomic and phenotypic characterization. PLoS One 4**:** e4982

Choi KH, Gaynor JB, White KG, Lopez C, Bosio CM, Karkhoff-Schweizer RR, Schweizer HP (2005) A Tn7-based broad-range bacterial cloning and expression system. Nat Methods 2**:** 443-448

Crépin S, Harel J, Dozois CM (2012) Chromosomal complementation using Tn7 transposon vectors in Enterobacteriaceae. Appl Environ Microbiol 78**:** 6001-6008

Datsenko KA, Wanner BL (2000) One-step inactivation of chromosomal genes in *Escherichia coli* K-12 using PCR products. Proc Natl Acad Sci U S A 97**:** 6640-6645

Demarre G, Guerout AM, Matsumoto-Mashimo C, Rowe-Magnus DA, Marliere P, Mazel D (2005) A new family of mobilizable suicide plasmids based on broad host range R388 plasmid (IncW) and RP4 plasmid (IncPalpha) conjugative machineries and their cognate *Escherichia coli* host strains. Res Microbiol 156**:** 245-255

Derbise A, Lesic B, Dacheux D, Ghigo JM, Carniel E (2003) A rapid and simple method for inactivating chromosomal genes in Yersinia. FEMS Immunol Med Microbiol 38**:** 113-116

Favre D, Viret JF (2000) Gene replacement in gram-negative bacteria: the pMAKSAC vectors. Biotechniques 28**:** 198-200, 202, 204

Gunn JS, Miller SI (1996) PhoP-PhoQ activates transcription of *pmrAB*, encoding a two-component regulatory system involved in *Salmonella typhimurium* antimicrobial peptide resistance. J Bacteriol 178**:** 6857-6864

Hoang TT, Karkhoff-Schweizer RR, Kutchma AJ, Schweizer HP (1998) A broad-host-range Flp-FRT recombination system for site-specific excision of chromosomally-located DNA sequences: application for isolation of unmarked *Pseudomonas aeruginosa* mutants. Gene 212**:** 77-86

Insua JL, Llobet E, Moranta D, Perez-Gutierrez C, Tomas A, Garmendia J, Bengoechea JA (2013) Modeling *Klebsiella pneumoniae* pathogenesis by infection of the wax moth *Galleria mellonella*. Infect Immun 81**:** 3552-3565

Lery LM, Frangeul L, Tomas A, Passet V, Almeida AS, Bialek-Davenet S, Barbe V, Bengoechea JA, Sansonetti P, Brisse S, Tournebize R (2014) Comparative analysis of *Klebsiella pneumoniae* genomes identifies a phospholipase D family protein as a novel virulence factor. BMC Biol 12**:** 41

Llobet E, Campos MA, Gimenez P, Moranta D, Bengoechea JA (2011) Analysis of the networks controlling the antimicrobial-peptide-dependent induction of *Klebsiella pneumoniae* virulence factors. Infect Immun 79**:** 3718-3732

Llobet E, Martinez-Moliner V, Moranta D, Dahlstrom KM, Regueiro V, Tomas A, Cano V, Perez-Gutierrez C, Frank CG, Fernandez-Carrasco H, Insua JL, Salminen TA, Garmendia J,

Bengoechea JA (2015) Deciphering tissue-induced *Klebsiella pneumoniae* lipid A structure. Proc Natl Acad Sci U S A 112**:** E6369-6378

Miller VL, Mekalanos JJ (1988) A novel suicide vector and its use in construction of insertion mutations: osmoregulation of outer membrane proteins and virulence determinants in *Vibrio cholerae* requires *toxR*. J Bacteriol 170**:** 2575-2583

Poirel L, Jayol A, Bontron S, Villegas MV, Ozdamar M, Turkoglu S, Nordmann P (2015) The *mgrB* gene as a key target for acquired resistance to colistin in *Klebsiella pneumoniae*. J Antimicrob Chemoth 70: 75-80
