# Supplementary material for: A Klebsiella pneumoniae antibiotic resistance mechanism that subdues host defences and promotes virulence
Source: EMBO Mol Med. 2017 Feb 15;9(4):430–47. doi: 10.15252/emmm.201607336 (PMC5376759; doi:10.15252/emmm.201607336)
Supplement: Supplementary file 2 — Expanded View Figures PDF [file EMMM-9-430-s002.pdf]

Expanded View Figures

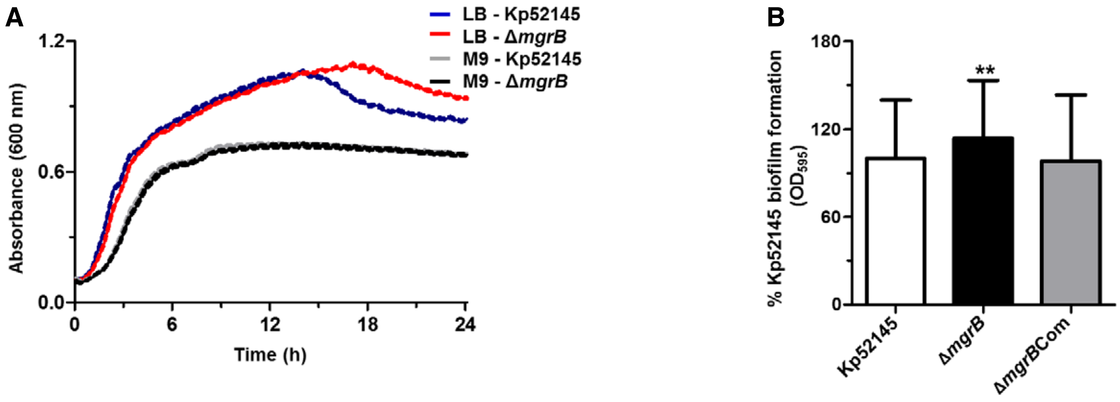

Figure EV1. Inactivation of *mgrB* in *K. pneumoniae* 52145 is not associated with an *in vitro* fitness cost.

A Growth kinetics of *K. pneumoniae* 51245 (blue and grey) and 52145-Δ*mgrB* strains (red and black) cultured in LB broth (LB) and 2% glucose M9 minimal media supplemented with thiamine and $MgSO_4$ (M9) over 24 h at 37°C. Values are presented as the mean ± SD of three independent experiments measured in triplicate.

B Short-term biofilm assay results for the *K. pneumoniae* 52145, 52145-Δ*mgrB* and 52145-Δ*mgrB*Com strains. Results are displayed as per cent biofilm relative to the wild-type strain with values presented as the mean ± SD of three independent experiments. Level of significance versus 52145 (***P* = 0.007) was determined using two-way unpaired *t*-test.

© 2017 The Authors

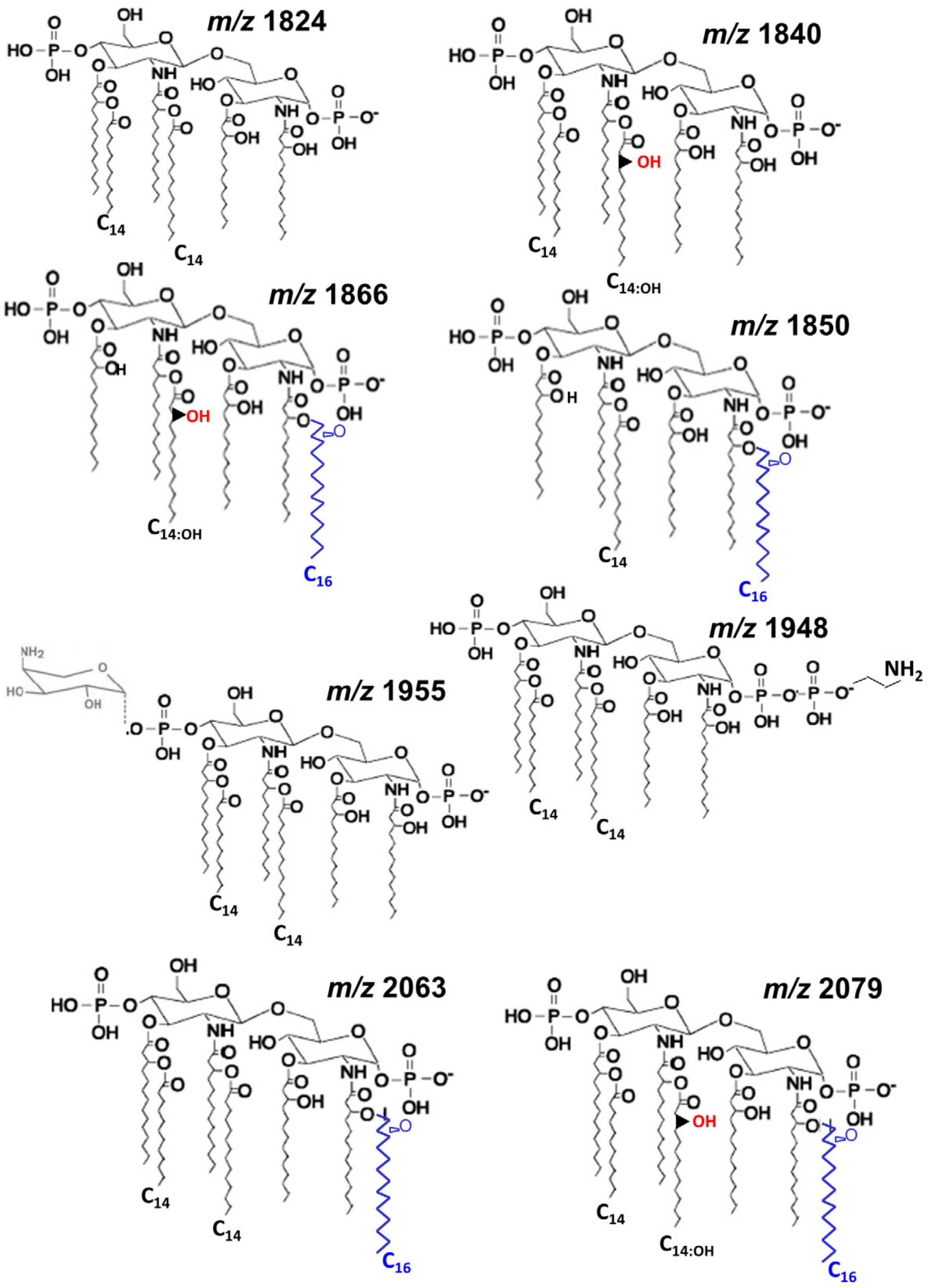

Figure EV2. Proposed lipid A chemical structures.

Proposed lipid A structures follow previously reported structures for *K. pneumoniae* (Sforza *et al*, 2004; Clements *et al*, 2007; Llobet *et al*, 2011, 2015) and other Gram-negative bacteria.

 © 2017 The Authors

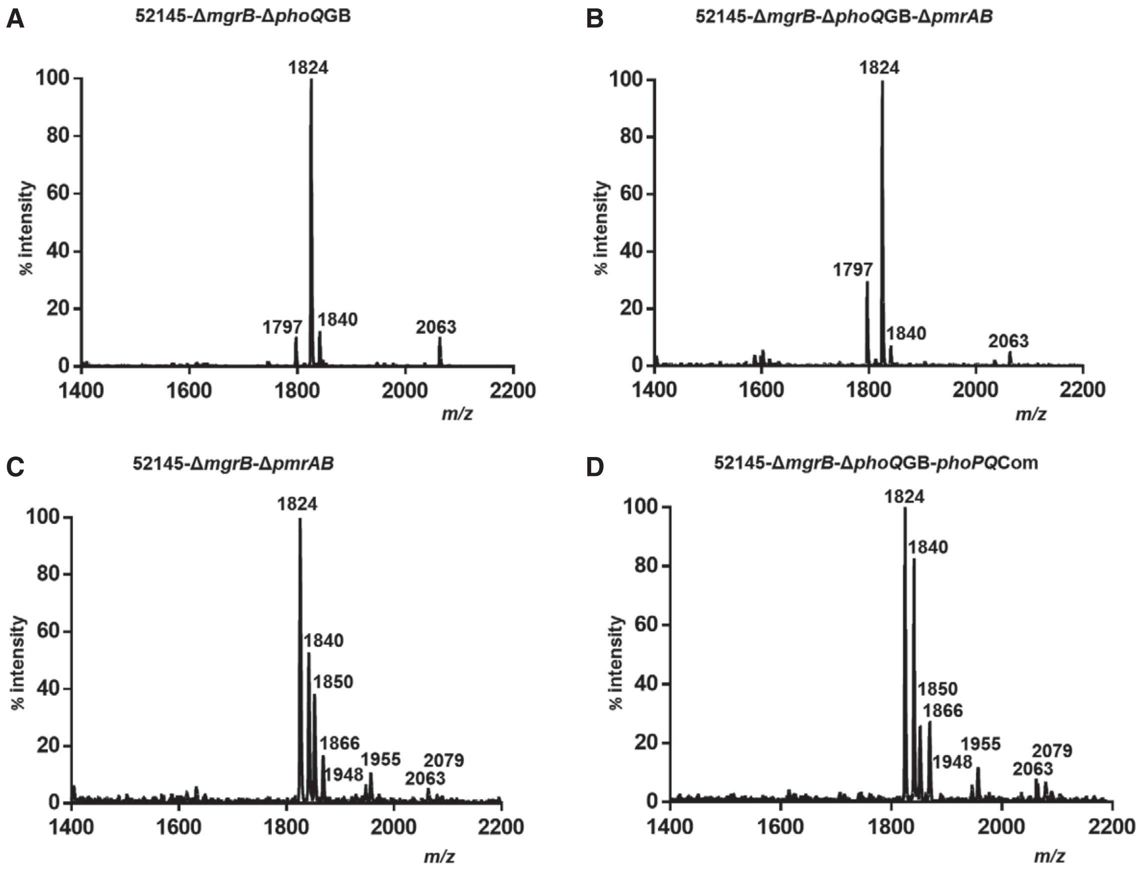

Figure EV3. Lipid A modifications in 52145-Δ*mgrB* occur in a PhoPQ-dependent manner.

A–D Negative ion MALDI-TOF mass spectrometry spectra of lipid A isolated from the *K. pneumoniae* 52145-Δ*mgrB*Δ*phoQ*GB (A), 52145-Δ*mgrB*-Δ*phoQ*GB-Δ*pmrAB* (B), 52145-Δ*mgrB*Δ*pmrAB* (C) and 52145-Δ*mgrB*-Δ*phoQ*GB-*phoPQ*Com (D) strains. Data represent the mass-to-charge (*m/z*) ratios of each lipid A species detected and are representative of three extractions.

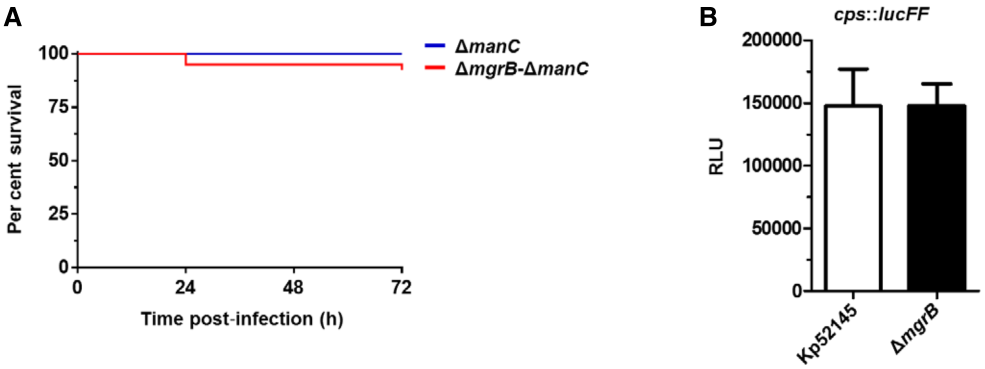

Figure EV4. Capsule polysaccharide (CPS) and MgrB each contribute to *K. pneumoniae* virulence in *G. mellonella*.

A Kaplan–Meier plot showing the per cent survival of *G. mellonella* over 72 h post-infection with 10^6 organisms of *K. pneumoniae* 52145-Δ*manC* (blue) and 52145-Δ*mgrB*-Δ*manC* (red). Forty larvae were infected in each group. Level of significance was determined using the log-rank (Mantel–Cox) test (α = 0.05).

B Activity of the *cps* promoter in *K. pneumoniae* 52145 and 52145-Δ*mgrB* carrying the *cps*::*lucFF* transcriptional fusion. Values (expressed in relative luminescence units [540 nm]) are presented as the mean ± SD of three independent experiments measured in triplicate. Level of significance was determined using two-way unpaired *t*-test.

© 2017 The Authors

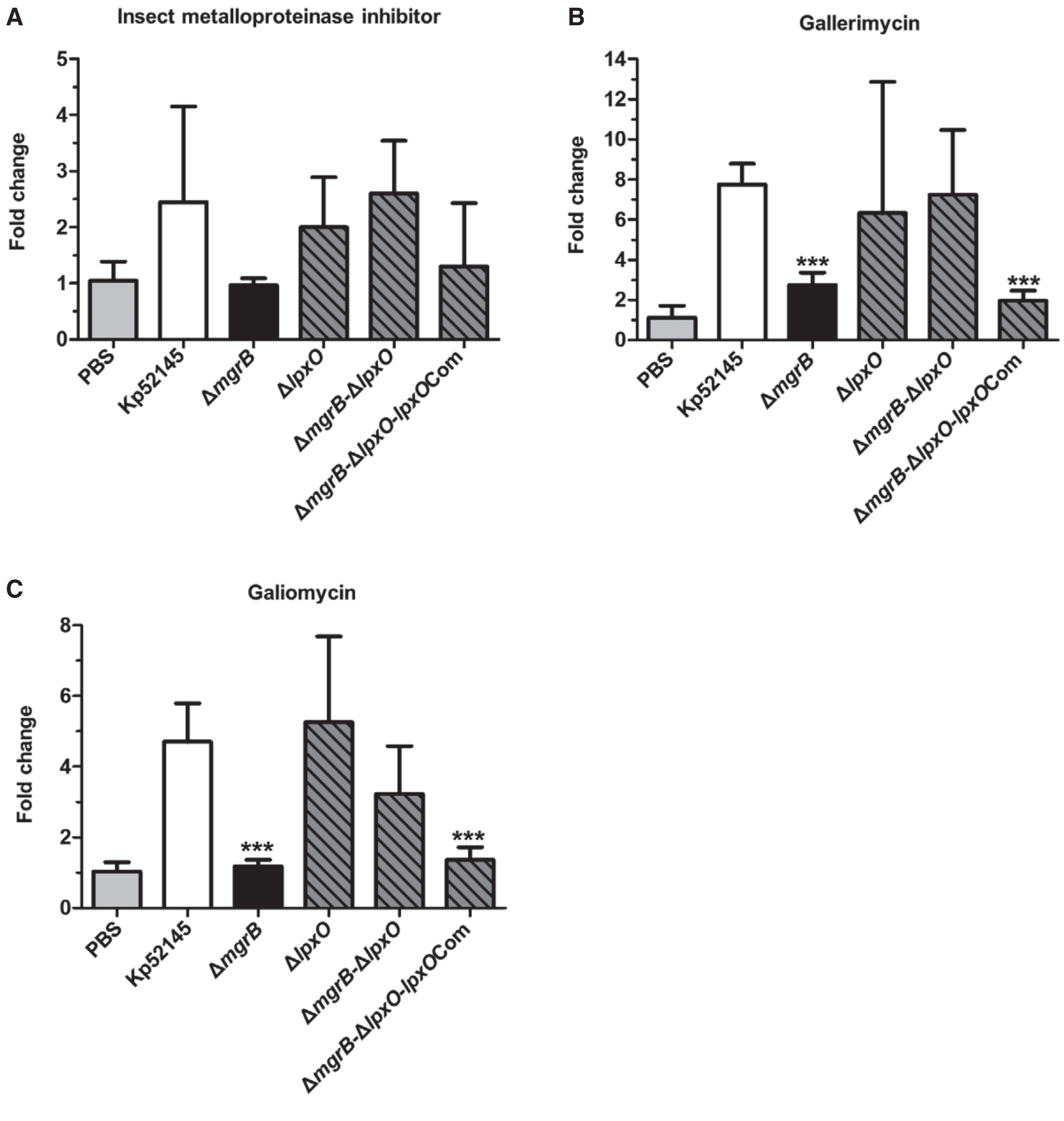

Figure EV5. Expression of insect metalloproteinase inhibitor, gallerimycin and galiomycin produced by *G. mellonella* during *K. pneumoniae* infection.

A–C *G. mellonella* antimicrobial peptide expression determined after 8 h of infection with *K. pneumoniae* 52145, 52145-Δ*mgrB*, 52145-Δ*mgrB*Com, 52145-Δ*lpxO*, 52145-Δ*mgrB*-Δ*lpxO* and 52145-Δ*mgrB*-Δ*lpxO*-*lpxO*Com by reverse transcriptase quantitative real-time PCR. Three larvae per group were infected, and values are presented as the mean ± SD of two independent cDNA preparations measured in duplicate. ****P* < 0.0001; versus 52145 determined using two-way unpaired *t*-test.

© 2017 The Authors
