# Supplementary figures and images for: A Klebsiella pneumoniae antibiotic resistance mechanism that subdues host defences and promotes virulence
Source: EMBO Mol Med. 2017 Feb 15;9(4):430–47. doi: 10.15252/emmm.201607336 (PMC5376759; doi:10.15252/emmm.201607336)

Figure 6A

I $\kappa$ B $\alpha$

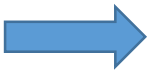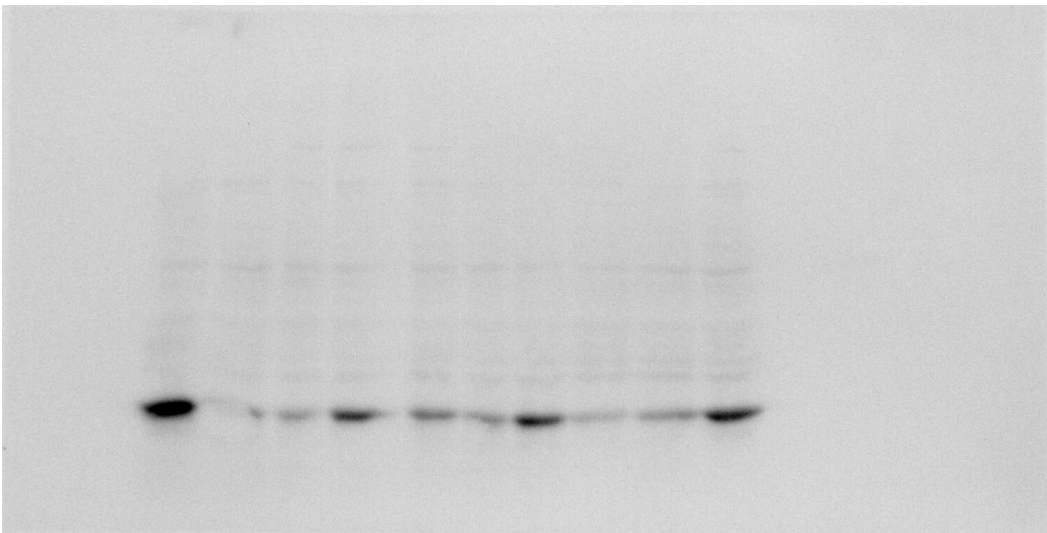

Tubulin

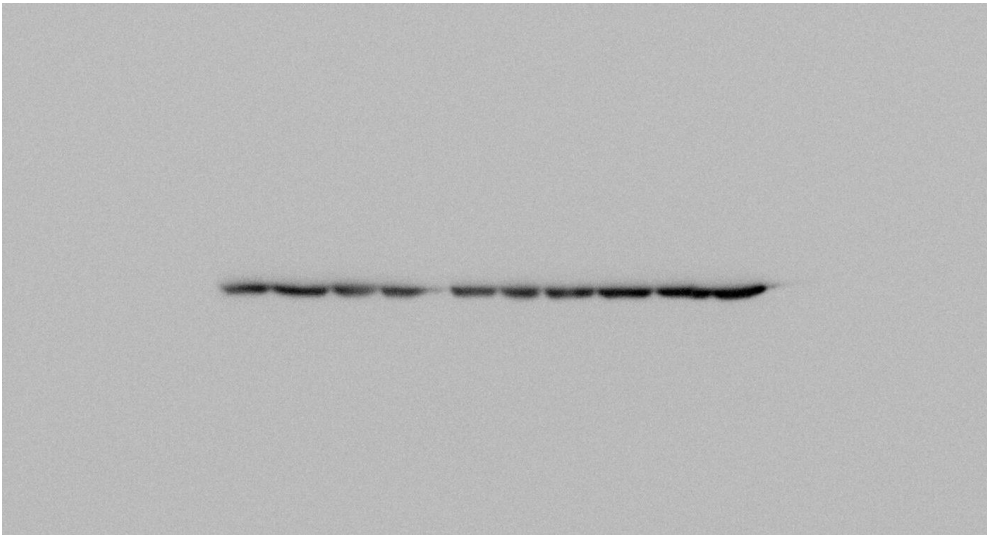

**Figure 6B**

**P-ERK**

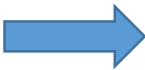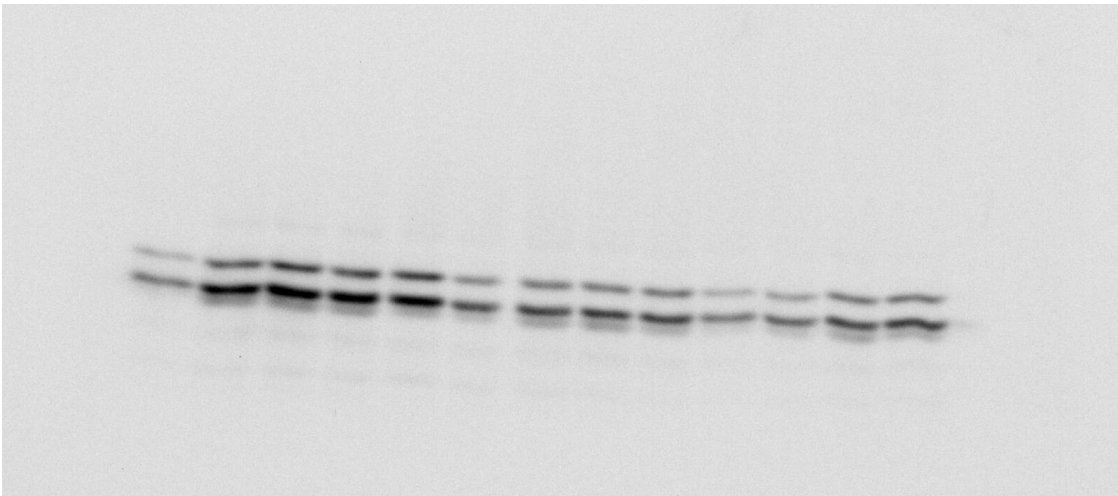

**P-p38**

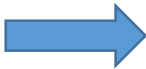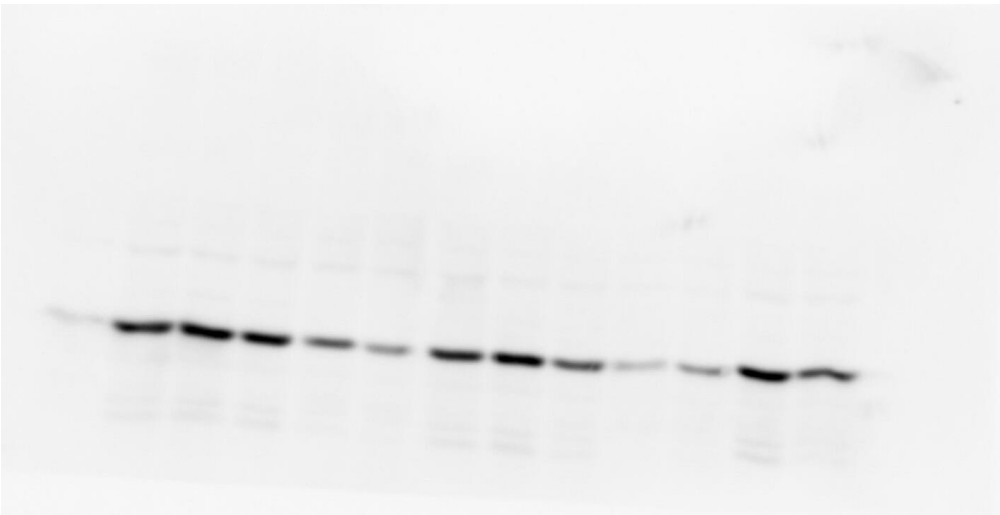

**P-JNK**

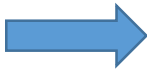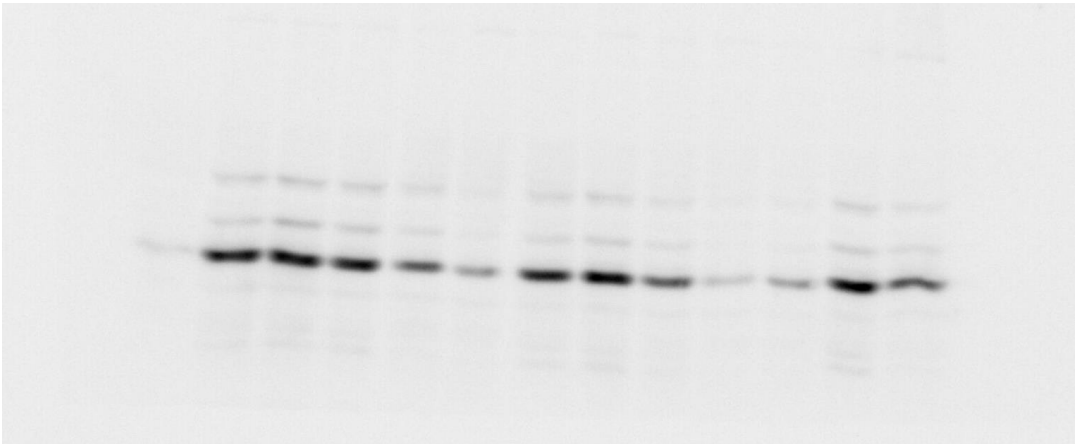

**Tubulin**

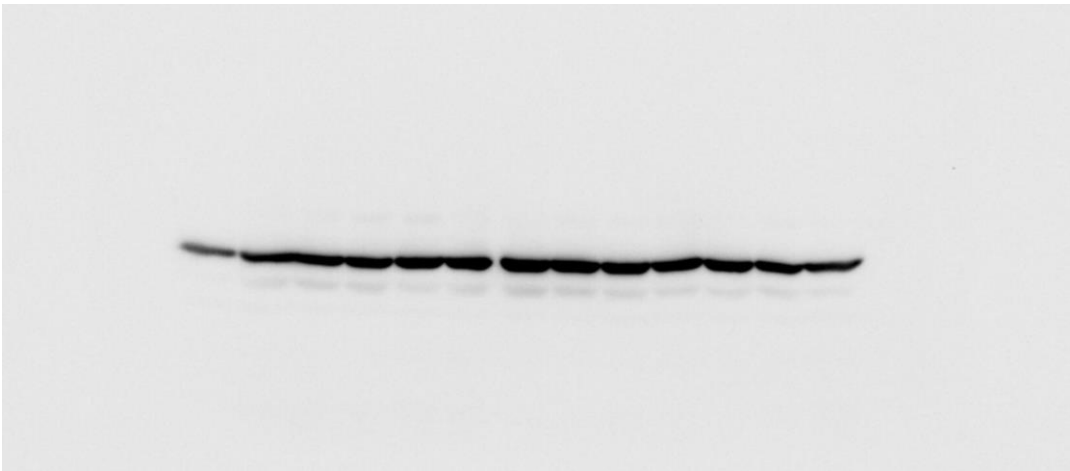

Figure 6C

P-JNK

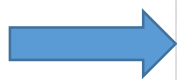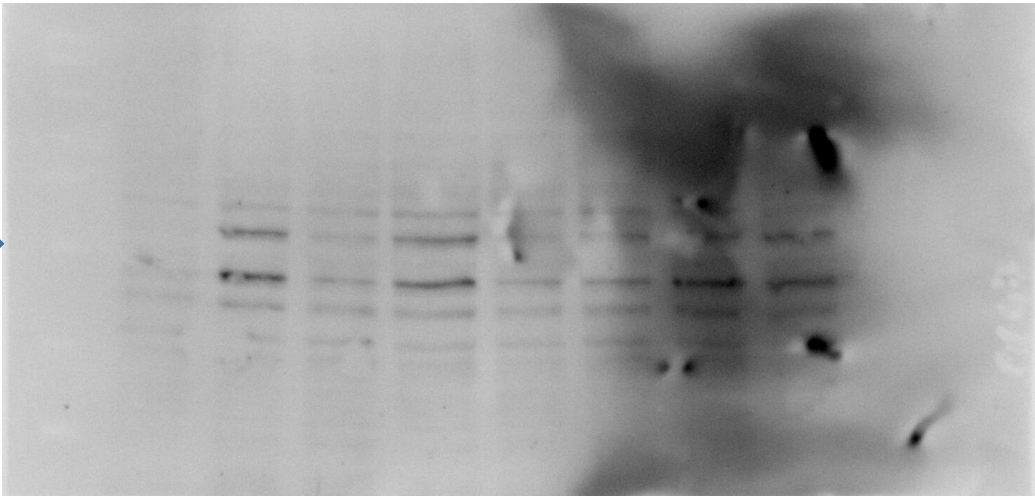

P-ERK

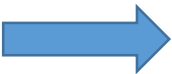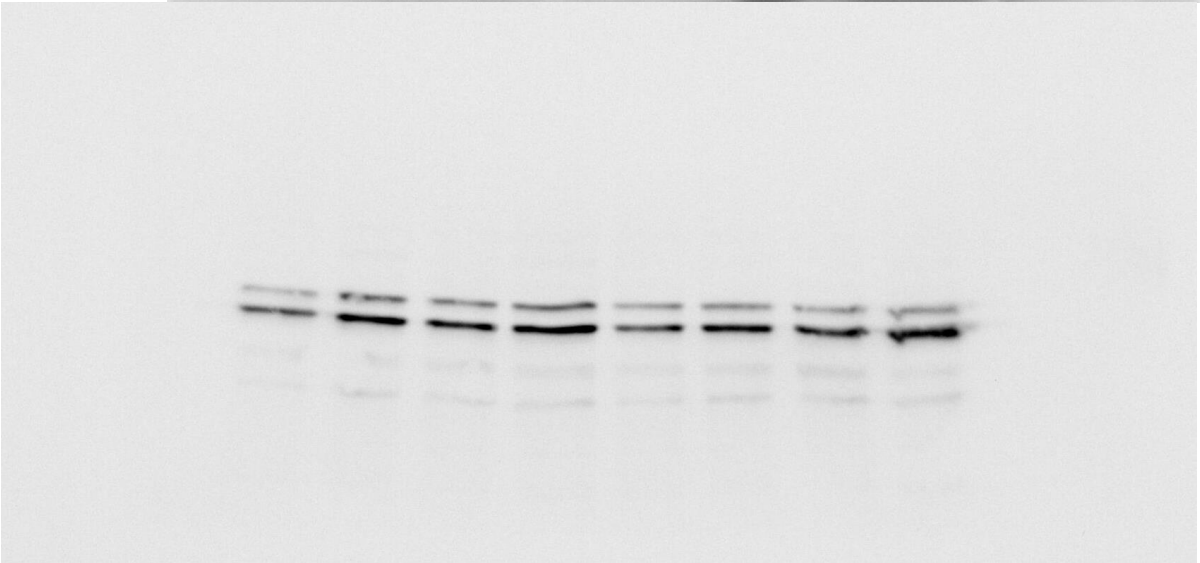

tubulin

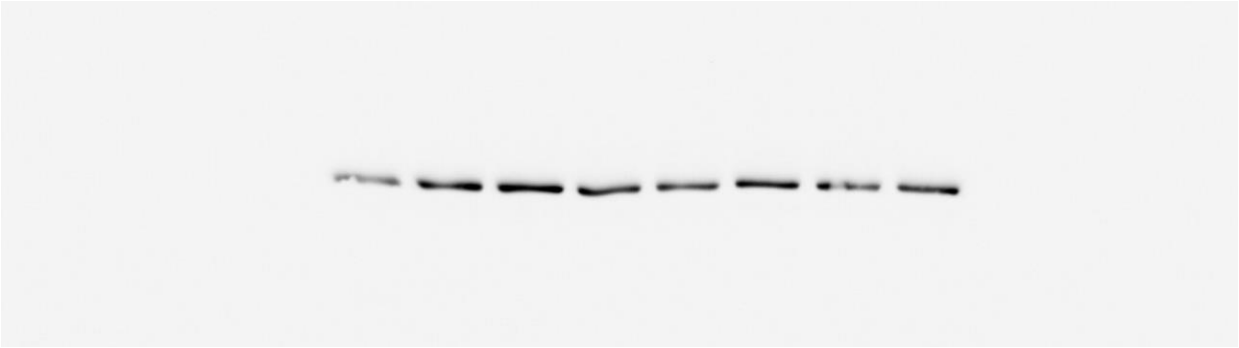

Supplement: Supplementary file 4 — Source Data for Figure 6 [file EMMM-9-430-s003.pdf]
